# Research on Anthocyanins from *Rubus* “Shuofeng” as Potential Antiproliferative and Apoptosis-Inducing Agents

**DOI:** 10.3390/foods12061216

**Published:** 2023-03-13

**Authors:** Fengyi Zhao, Huifang Zhao, Wenlong Wu, Weifan Wang, Weilin Li

**Affiliations:** 1 Institute of Botany, Jiangsu Province and Chinese Academy of Sciences (Nanjing Botanical Garden Mem. Sun Yat-Sen), Jiangsu Key Laboratory for the Research and Utilization of Plant Resources, Qian Hu Hou Cun No. 1, Nanjing 210014, China; zhaofengyi92@163.com (F.Z.); nuzhenzi_1984@163.com (H.Z.); 2 College of Chemical Engineering, Nanjing Forestry University, Nanjing 210037, China; 3 Co-Innovation Center for the Sustainable Forestry in Southern China, College of Forestry, Nanjing Forestry University, Nanjing 210037, China; wlli@njfu.edu.cn

**Keywords:** blackberry, anthocyanin, antiproliferative activity, apoptosis, DNA binding

## Abstract

Blackberries have high nutritional value and strong biological activities, such as antiproliferative activity. Anthocyanins are important functional components in blackberries. We collected 25 kinds (lines) of blackberries from our nursery to investigate antiproliferative agents in natural foods. Among them, the Shuofeng variety had the highest anthocyanin content, with 2.54 mg/g of fresh fruit, which increased to 357.75 mg/g of dried powder through ultrasound-assisted solvent extraction and macroporous resin adsorption. Additional experiments showed that Shuofeng’s anthocyanin content had high anti-HepG2 activity in vitro and in vivo, as well as activity against Hela (68.62 μg/mL), HepG2 (55.85 μg/mL), MCF-7 (181.21 μg/mL), and A549 cells (82.01 μg/mL), as determined by MTT assay. It also had no apparent toxic effects. The combination of DDP and DOX significantly enhanced the antiproliferative activity of the four cell lines. The IC_50_ value of Shuofeng’s anthocyanin content combined with DOX in HepG2 cells was the lowest at only 0.08 μg/mL, indicating that the combination of drugs had additive and synergistic effects. Shuofeng’s anthocyanin content might intercalate into DNA and alter or destroy DNA, causing apoptosis and inhibiting cell proliferation. Our results show that blackberry anthocyanins can inhibit the proliferation of cancer cells and their possible mechanisms. However, we must study the deeper mechanism and explore its targeting effects in the future.

## 1. Introduction

Blackberry (*Rubus* spp.) is a type of thorny shrub that grows aggregate fruit and is a member of the rose family [1]. It is an important fruit that is widely cultivated in Europe and North America, with large-scale planting occurring in China since the 1980s [2]. Blackberry is a third-generation, new special fruit recognized by the United Nations Food and Agriculture Organization due to its rich nutrients and medicinal properties. Known as “life fruit”, it is popularly consumed in fresh or processed forms [3,4]. This berry has one of the highest contents of anthocyanins, flavanol glycosides, and other phenols among common fruits and vegetables, contributing to its strong biological potential, including antioxidant, antiaging, anti-inflammatory, antibacterial, and anticancer activities [5,6,7,8,9].

Polyphenols and polyphenol-rich diets are well recognized due to their role in preventing many diseases [10]. Anthocyanins are secondary metabolites generated by the synthesis pathways of the phenylpropionic acid and flavonoids in plants [11]. There are six major anthocyanidins in plants: cyanidin, delphinidin, petunidin, pelargonidin, peonidin, and malvidin [12]. They are commonly linked by glycosidic bonds with one or more glucose, rhamnose, arabinose, xylose, or galactose molecule. One or more of the ferulic, coumaric, caffeic, p-hydroxybenzoic, and other aromatic and fatty acid molecules in anthocyanins react with glycosides and hydroxyl groups to form acylated anthocyanins. There are more than 500 types of anthocyanins known in nature. In recent pharmacological studies, anthocyanins have demonstrated great physiological activities by improving microcirculation, promoting retinoid resynthesis, demonstrating anticancer and anti-inflammation effects [13], and reducing blood glucose and lipid levels [14,15]. Polyphenol-rich fruits have demonstrated strong chemopreventive and anticarcinogenic effects against different types of cancer in both in vitro and in vivo studies [16,17,18].

Cancer, which is caused by malignant tumors, seriously threatens human life and health. Modern tumor treatments include surgery, chemotherapy, and radiotherapy, but these treatments are traumatic to the human body and have severe side effects. Anthocyanins have attracted researchers’ attention as a natural plant pigment because of their various biological activities, especially their anticancer properties [19]. Seeram et al. found that blackberry extracts could inhibit the proliferation of human oral (KB and CAL-27), colon (HT-29 and HCT116), breast (MCF-7), and prostate (LNCaP) tumor cell lines in a dose-dependent manner [20]. Methyl jasmonate treatment significantly increased the total anthocyanin and phenol contents in blackberry fruits; inhibited cell proliferation in human lungs, A549 cells, and HL-60 leukemia cells; and induced the apoptosis of HL-60 cells [21]. Cyanidin 3-O-glucoside (C3G) may increase cisplatin’s antitumor activity to reduce the adverse effects of chemotherapy in cervical cancer [22]. Several mechanisms have been proposed to mediate the anticancer properties of anthocyanins. For example, anthocyanins in blackberry are thought to regulate cell proliferation by altering the cell signaling pathways that activate AP-1 (activator protein 1) and NFkB (nuclear factor kB) [23]. C3G treatment promotes apoptosis in Hela cells by downregulating the PI3K/AKT/mTOR signaling pathway; activating Bax, p53, and TIMP-1 (tissue inhibitor of metalloproteinase-1); decreasing cyclin D1 and Bcl-2 expression; and blocking the cell cycle at the G1 phase [24]. Delphinidin and cyanidin inhibit platelet-derived growth factor (PDGF) (AB)-induced vascular endothelial growth factor (VEGF) expression in vascular smooth muscle cells by decreasing the p38 mitogen-activated protein kinase (MAPK) and c-JUN NH2-terminal kinase (JNK) signaling pathways [25]. Berry anthocyanins act on the β-catenin, Wnt, and Notch pathways to synergistically inhibit the growth and proliferation of human non-small cell lung cancer cells and their downstream target proteins [26].

**Scheme 1:**
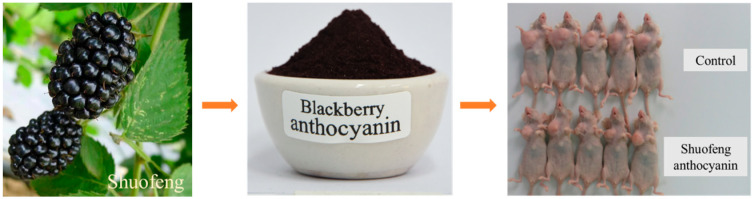
The creative idea of this article.

Although anthocyanins have been proposed to exert cancer-specific cytotoxic activity against cancer cells, they have shown minimal to no toxicity against normal cells [17]; thus, the discovery of more antiproliferative agents from natural foods is needed. In this work, we collected 25 kinds (lines) of blackberries and investigated their anthocyanin contents. We also extracted and purified “Shuofeng” blackberry, which had the highest anthocyanin content. Then, we explored its inhibition of cancer cell proliferation both in vitro and in vivo, the effect of combined drugs, and the possible mechanism (Scheme 1). We hope to provide a theoretical basis for developing blackberry’s additional value and for exploring more natural foods.

## 2. Materials and Methods

### 2.1. General Materials

We collected 25 kinds (lines) of blackberries from the Institute of Botany, Jiangsu Province, and the Chinese Academy of Sciences, Baima field. Analytical reagents (AR) and solvents were used in general experimental procedures unless otherwise stated. Purification was conducted by flash chromatography with microporous resin (LS-300B). UV–vis adsorption spectra were recorded using a TU-1810 spectrophotometer.

Other equipment: EX-200A Electronic Balance, ZD-2 Automatic Potential Titrator, WP 700TL23-k5Galanz Microwave Oven, PL-5-B Low-speed Centrifuge, Philips Blender HR2838, Agilent 1260UHPLC-6530 Q-TOF MS, NIB610 microscope, KQ-300DE ultrasonic cleaner, BD Accuri C6 flow cytometry, and Zeiss LSM 900 Laser Scanning Confocal Microscope.

### 2.2. Soluble Solids Content

We measured the soluble solid contents of 25 kinds of blackberries (fresh fruit) using Pal-1 (unit: °Brix).

### 2.3. Titratable Acid Content Determination

According to GB/T12456-2008, “Method for Determination of Total Acid in Food”, the total acid results were calculated with citric acid. We crushed 100 g of fresh blackberry, deposited 3 g into a 50 mL centrifuge tube, and added 20 mL of deionized water. The mixture underwent ultrasonic treatment at 40 °C and 60 Hz for 30 min and was centrifuged at 5000× *g* rpm for 5 min. We transferred the supernatant to a 100 mL beaker and measured the initial pH value of the solution with a pH meter. The NaOH standard solution (0.1 mol/L) was titrated until the pH value of the solution was 8.0, which was the endpoint. We recorded the amount of NaOH and calculated the titratable acid content.

### 2.4. Standard Curve Drawing

We established the standard curve of C3G for the test determining anthocyanin content in Table 1 using the following methods: standard stock solution (1 mg/mL): 0.005 g ± 0.001 g of C3G standard solution was dissolved in a small amount of methanol (500 µg/mL), transferred into a 10 mL flask, kept at a constant water temperature, and shaken to prepare 500 µg/mL C3G standard solution. The standard solution was then diluted to 50, 75, 100, 125, and 150 µg/mL. We deposited 300 μL into a 10 mL centrifuge tube and added 2.7 mL of buffer at pH 1.0, which was shaken and stored in the dark at room temperature for 20 min. The buffer was used as a blank control. Then, we measured absorbance value A at 510 nm with a 1 cm colorimeter and three times in parallel to draw a standard curve. The linear regression equation between the mass concentration of C3G (µg/mL) and absorbance A was y = 0.0455x − 0.0881; the correlation coefficient was R^2^ = 0.9996, indicating the good linear relationship between the mass concentration of C3G and absorbance A.

### 2.5. Extraction

We referred to and improved upon Zhao’s article [27] by thawing 3 kg of frozen Shuofeng blackberry fruits, hydrolyzing them with 0.02% pectinase at 40–50 °C for 2 h, and centrifuging them at 4000× *g* rpm for 5 min. We extracted the precipitates with 70% ethanol (containing 0.1% formic acid by volume). After ultrasound-assisted extraction at 40 °C and 60 Hz for 30 min, the extract was centrifuged at 5000× *g* rpm for 5 min.

### 2.6. Purification

We referred to and improved upon Zhao’s article [28] by evaporating the ethanol from extract, retaining the aqueous solution, and adsorbing it with a macroporous resin column. The solvent was eluted with 70% ethanol. We collected the eluent, repeated the adsorption, and freeze-dried the solvent. The final product was S^1^.

### 2.7. Cell Culture, Antiproliferative Activities, and Cytotoxicity Assay

Reagents and the compounds MTT, DMEM, FBS, and penicillin/streptomycin were all commercially purchased from Nanjing Keybionet Biotechnology Co., Ltd. (Nanjing, China). Hela, HepG2, MCF-7, A549, and HUVEC cells were purchased from the National Collection of Authenticated Cell Cultures.

We added cancer cells to 96-well plates with a density of 1 × 10^4^ cells per well. The culture media were removed after 12 h of incubation at 5% CO_2_ and 37 °C, and the cells were incubated with DDP, DOX, S^1^-DDP, and S^1^-DOX. The standard sample was dissolved in DMEM at different concentrations (each concentration repeated three times) for 48 h at 5% CO_2_ and 37 °C. Afterward, we removed the culture media and added the new culture medium containing MTT (1 mg/mL), followed by incubation for 4 h. After removing the medium, we added 200 μL of DMSO to each well to dissolve the formazan crystals. Absorbance was measured at 595 nm in a microplate photometer. Cell viability values were determined (at least three times) according to the following formulae: cell viability (%) = the absorbance of the experimental group/the absorbance of the blank control group × 100% [29,30].

### 2.8. Induction of Apoptosis Assay

The apoptosis induction assay was operated using a BD Accuri C6 flow cytometry instrument and Annexin V-FITC/PI from Shanghai Beyotime Biotechnology Co., Ltd. (Shanghai, China) in China.

We also investigated whether S^1^ could induce apoptosis using DMSO as a negative control. HepG2 cells (1 × 10^6^) were cultured in 35 mm dishes and incubated for 24 h at 37 °C. The cells were incubated with DMSO (5 μg/mL) and S^1^ (12.5, 25, and 50 μg/mL) for 48 h (each concentration was repeated three times for optimal incubation time), washed, trypsinized (non-EDTA), and centrifuged (2000× *g* rpm/min). Cells were harvested and resuspended in 500 μL buffer containing Annexin V-FITC (5 μL of Annexin V-FITC and 5 μL of PI). The cells were stained with Annexin V-FITC and incubated for 5–15 min in the dark. Cells (1 × 10^4^) were collected for flow cytometry analysis with a single 488 nm argon laser.

### 2.9. Detection of Intracellular Reactive Oxygen Species (ROS)

The effect of S^1^ on ROS production was determined by BD Accuri C6 flow cytometry. After overnight culture in 6-well plates, HepG2 cells were incubated with S^1^ (0 and 25 μg/mL) for 48 h, followed by dihydrodichlorofluorescein diacetate (DCFH-DA) (10 μM) for 30 min at 37 °C in the dark. Finally, the cells were collected and washed with PBS. The samples were analyzed using flow cytometry. Excitation wavelengths = 488 nm; emission wavelengths = 535 nm.

### 2.10. Confocal Fluorescence Images

HepG2 cells were cultured in 35 mm glass dishes for 24 h and incubated with S^1^ (25 μg/mL) at 5% CO_2_ and 37 °C for 48 h before fluorescence imaging. After incubation, the medium was removed, and the cells were washed with PBS (pH = 7.4) and incubated with fresh medium. We examined the confocal fluorescence images via the excitation of Annexin V and PI channels.

### 2.11. In Vivo Experiment

Our in vivo experiment was provided by Nanjing Keygen Biotech. Co., Ltd., China. A tumor model was established by the subcutaneous injection of 1 × 10^6^ HepG2 cells into the right axilla of each Balb/C nude mouse. Subsequently, HepG2 tumor-bearing mice were divided into two groups (five mice per group) for the experiment: (1) PBS (control group) and (2) S^1^. When the tumor volume reached 80 mm^3^, PBS and S^1^ solution (dose = 15 mg/kg) were injected intravenously. The tumor size of each mouse in our experiment was measured with a vernier caliper every 3 days for the next 25 days. To accurately evaluate tumor growth inhibition, mice were sacrificed after 25 days and tumor tissues were collected, photographed, and weighed. On the 25th day, we collected the tumor, heart, kidney, liver, lung, and spleen tissues from the mice in each group for HE staining and pathological examination. Tumor size was calculated as volume = 0.5 × (tumor length) × (tumor width)^2^. We calculated the tumor growth inhibition rate according to the following formula: inhibition efficiency (%) = (1 − the weight of the experimental group/the weight of the control group) × 100%.

CD31 immunohistochemical staining was performed on the mice after intravenous injection of S^1^ (15 mg/kg) and PBS control on the 25th day.

### 2.12. DNA Binding Modes

We added 3 mL of DNA-EB mixture (C_DNA_/C_EB_ = 10) to the sample pool and equal volumes of composite samples each time to increase the concentration ratio of S^1^ to DNA. Fluorescence spectra were determined at an excitation wavelength of 520 nm.

### 2.13. Data Analysis

The analytical data were statistically analyzed using SPSS Statistics 16.0 (IBM, Chicago, IL, USA). The data obtained were statistically managed using one-way ANOVA at a 0.05% level of reliability. Origin 2019b (OriginLab Crop., Northampton, MA, USA) was used to analyze the IC_50_ value with linear fitting. All experiments were repeated three times and the results represented the mean ± standard deviation.

## 3. Results

### 3.1. Main Nutrients Analysis

We investigated 25 kinds (lines) of blackberries along with their nutritional ingredients, including soluble solids, organic acids, solidity–acid ratio, and total anthocyanin content (Table 1).

Usually, the higher the sugar content, the higher the soluble solids and the sweeter the fruit. Table 1 shows that the average soluble solid content was 11.13%, and the content of 15-1-11 was the highest at 14.43%, followed by 15-1-1 (13.17%), 15-1-2 (12.63%), 16n-12 (12.63%), 15-1-10 (12.50%), etc. The more acidic the fruit, the higher the total acid content. Out of these blackberry varieties, Chester had the highest total acid content, with 1.24%, followed by Zaohei (1.22%), 15-1-2 (1.15%), 15-1-11 (1.12%), 10-5n-2 (1.05%), etc. We obtained the solid acid ratio to evaluate fruit taste through the ratio of soluble solids to total acid content. A higher solid–acid ratio suggests that the fruit is sweeter and has a higher sugar content. Our results showed that 16n-12 was the sweetest, with the highest solid acid ratio of 16.93, followed by 15-1-17 (16.18), 15-1-1 (15.80), Ningzhi‘2’ (15.27), Commanche (14.55), etc. Blackberry has a significant amount of anthocyanin, the main factor behind its biological activity. It can also be said that anthocyanin content is important to the quality of blackberries. Based on total anthocyanin content, Shuofeng was the highest at 2.54 mg/g. The other top five varieties of blackberries were 16n-12 (2.35 mg/g), 15-1-1 (2.29 mg/g), 15-1-2 (2.29 mg/g), and Shawnee (2.25 mg/g).

### 3.2. Extraction and Purification

Many researchers in China and abroad have characterized the macroporous resin adsorption method as a method to purify anthocyanins that has high efficiency, stable quality, low cost, and simple operation [28]. The stability of anthocyanins is often poor. Considering that blackberry anthocyanins are exposed to air for a long time, light, temperature, and oxidants will affect them. The resin’s adsorption time must be controlled within 5 h during the purification process. After extracting the fresh fruit and pomace from Shuofeng blackberries, the anthocyanin content was 2.54 mg/g. With further purification, the anthocyanin content of the dried powder increased to 357.75 mg/g. Blackberries in the domestic market do not have such a high anthocyanin content. To our knowledge, the highest anthocyanin content on the domestic market is the Lanmei 1 blueberry, with 443.08 mg/g [31]. Anthocyanins from Shuofeng blackberries can be developed as a natural food product with added value.

### 3.3. Component Analysis

**Figure 1:**
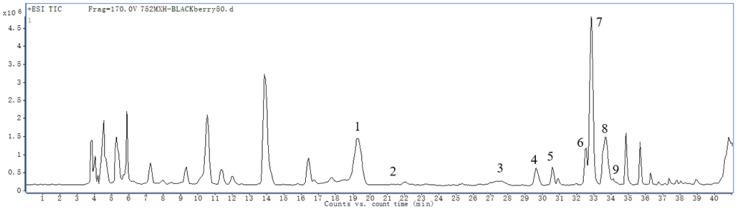
UPLC-QTOF-MS total ion flow diagram of anthocyanin components in blackberries.

We used ultra-performance liquid chromatography (UPLC) coupled with quadrupole time-of-flight (Q-TOF) mass spectrometry (MS) (UPLC-QTOF-MS) to analyze S^1^. Our results showed that the main anthocyanin components were cyanidin and pelargonidin, glycosylated or acylated forms of glucose, galactose, and arabinose (Figure 1, Table 2). The molecular ion [M+H]^+^ is *m*/*z* 449.1 and the fragment ion is *m*/*z* 287.1, corresponding to the molecular weight of cyanidin, which is obtained by the molecular ion’s loss of a neutral fragment (glucoside) with a mass number of 162. Cyanidin 3-O-glucoside is considered the most common and abundant anthocyanin in blackberries. The molecular ion [M+H]^+^ is *m*/*z* 419.1 and the fragment ion is *m*/*z* 287.1, which is formed by *m*/*z* 419.2 losing a neutral fragment with a mass number of 132. The latter has the same mass number as five-carbon sugars (arabinoside or xyloside) and cyanidin after losing a water molecule. The molecular ion [M+H]^+^ is *m*/*z* 535.1 and the fragment ionization is *m*/*z* 287.1, which loses a neutral fragment with a mass number of 248. After hydrolyzing blackberry anthocyanins, only glucosides and arabinosides form glycogroups. Therefore, the glycogroup in this neutral fragment may be composed of glucoside or arabinoside. In the case of glucoside, the acylation of glucoside (mass number 162) and malonic acid (mass number 86) may form the neutral fragment. If arabinoside forms a neutral fragment of 248, it may acylate with malic acid (mass number 116). There have been no reports of malate acylation in blackberry anthocyanins. Therefore, cyanidin’s 3-O-malonyl glucoside is inferred along with the others presented in Table 2. Since blackberries are the only fruit to contain cyanidins, it is easier to authenticate blackberry products because grapes or strawberries contain more anthocyanins.

### 3.4. Biological Evaluation

#### 3.4.1. Antiproliferative Activities

We compared the antiproliferative activities of S^1^ to the well-known antiproliferative drugs DDP and DOX, S^1^-DDP (S^1^ combined with cis-platinum), S^1^-DOX (S^1^ combined with doxorubicin), and standard sample (cyanidin-3-O-glucoside). We evaluated them on the growth of human cancer cell lines Hela, HepG2, MCF-7, A549, and the normal cell HUVEC in vitro by MTT assay. DDP and DOX were used as the positive control. Table 3 shows our results.

The toxicity of S^1^ was lower than DDP, DOX, S^1^-DDP, and S^1^-DOX against HUVEC cells. For Hela cells, the combined effect of S^1^ and DDP or DOX was more effective than individual incubation according to the IC_50_ values of S^1^, S^1^-DDP, S^1^-DOX, DDP, DOX, and C3G, which were 68.62 μg/mL, 32.70, 2.88, 18.50, 0.96, and 126.31 μg/mL, respectively. For HepG2 cells, S^1^ combined with DOX had the highest anti-HepG2 activity, with the lowest IC_50_ value of 0.08 μg/mL. Furthermore, S^1^ and DOX, and S^1^ combined with DDP also had higher activity than S^1^, suggesting that the combination of DDP or DOX may have additive and synergistic effects. Both S^1^ combined with DOX (0.80 μg/mL) and DDP (118.13 μg/mL) had higher activity against MCF-7 cells than S^1^ (181.21 μg/mL). The higher anti-A549 activity of S^1^ combined with DDP and DOX had a lower IC_50_ value of S^1^-DDP (68.47 μg/mL) and S^1^-DOX (0.44 μg/mL) than S^1^ (82.01 μg/mL).

Our results revealed that combined drugs can enhance treatment effects due to the increased antiproliferative activity with S^1^ and the combination of DDP or DOX. Among them, S^1^ combined with DOX (0.08 μg/mL) had the lowest IC_50_ value against HepG2 cells, suggesting that this combination may be a potential agent against HepG2 cells.

#### 3.4.2. Induction of Apoptosis

The apoptosis assay can provide important information on the action mode of cells. In our previous work, we found that S^1^ had high anti-HepG2 activity. We continued studies into whether S^1^ anthocyanin could induce apoptosis in HepG2 cells.

**Figure 2:**
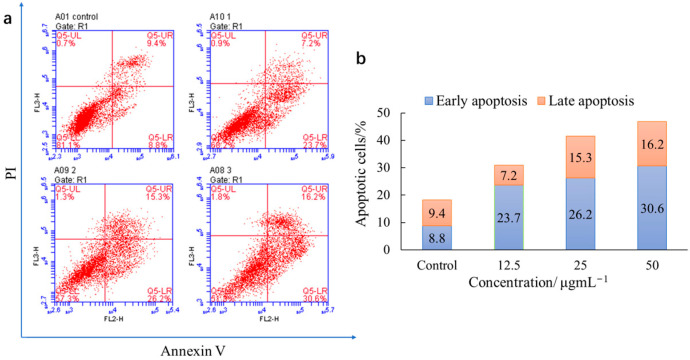
An apoptosis assay of HepG2 cells treated with S^1^. (**a**) Control was HepG2 cells treated with DMSO (negative control), followed by S^1^ at 12.5, 25, and 50 μg/mL; (**b**) apoptotic rate of control and S^1^ at 12.5, 25, and 50 μg/mL, including early apoptosis and late apoptosis.

Figure 2 shows that living cells reduced and apoptotic cells increased with the incubation of S^1^ and HepG2 cells at the concentrations of 12.5, 25, and 50 μg/mL, indicating a dosage dependence. The early apoptotic rate was 23.7, 26.2, and 30.6%. The late apoptotic rate was 7.2, 15.3, and 16.2% when S^1^ was 12.5, 25, and 50 μg/mL, respectively. In the apoptosis-inducing process, living cells become apoptotic cells with increased concentrations. These results suggest that S^1^ inhibits cell proliferation by inducing apoptosis.

#### 3.4.3. Detection of Intracellular Reactive Oxygen Species (ROS)

Sufficient evidence revealed that apoptosis induction in cancer cells could be mediated by reactive oxygen species (ROS). ROS are the potential cause of many chronic diseases that enhance intracellular oxidative stress [32].

**Figure 3:**
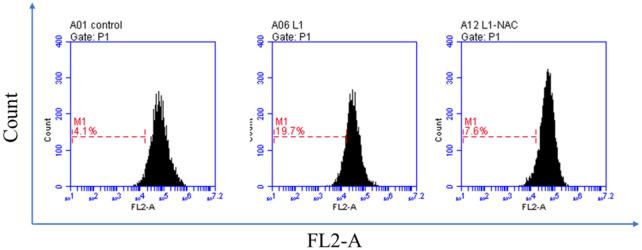
ROS generation assay of compound S^1^ against HepG2 cells. HepG2 cells not treated with S^1^ were used as a control for 48 h and treated with S^1^ (25 μg/mL). NAC was added after being treated with S^1^ (25 μg/mL) for 48 h. ROS production was measured by flow cytometry.

We used flow cytometry to determine the effects of S^1^ on the cellular ROS level of HepG2 cancer cells. S^1^ (25 μg/mL) was incubated with HepG2 cells for 48 h, as shown in Figure 3. The percentage of ROS was 19.7%, whereas the control was 4.1%. The percentage was back to 7.6% after adding the NAC, also known as an ROS inhibitor. Generally, these data indicated that S^1^ induced ROS production in HepG2 cells and led to cell death.

#### 3.4.4. Confocal Fluorescence Images

**Figure 4:**
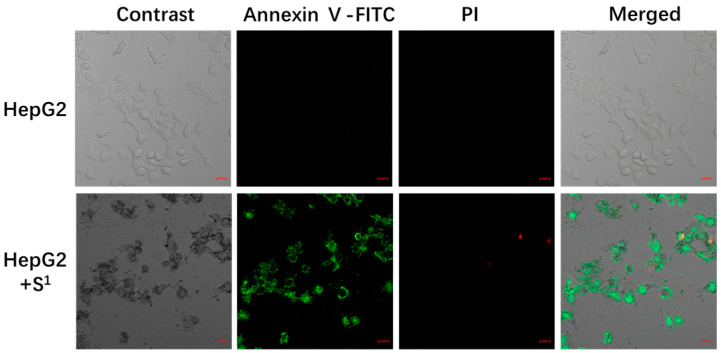
Confocal fluorescence images of S^1^ (25 μg/mL) incubated with HepG2 cells for 48 h at 37 °C. Scale bars are 20 μm.

We made the apoptosis-inducing process visible to prove that S^1^ inhibits cell proliferation through confocal fluorescence images (Figure 4).

The nucleus was stained red with propidium iodide (PI); the cytoplasm was stained green with Annexin V-FITC. Normal cells were not stained with fluorescence, apoptotic cells were stained with green fluorescence only, and necrotic cells were stained with green and red fluorescence. The negative control cells had almost no green or red fluorescence signals, suggesting that the cells were in a normal state. After incubation at 37 °C for 48 h, S^1^ (25 μg/mL) had a strong green fluorescence signal and a weak red fluorescence signal in the cytoplasm, suggesting that S^1^ can induce apoptosis.

#### 3.4.5. In Vivo Experiment

For further investigation, we injected S^1^ into the mice during the in vivo experiment. We intravenously injected S^1^ (15 mg/kg) into tumor mice with HepG2 cells every 3 days for 25 days.

**Figure 5:**
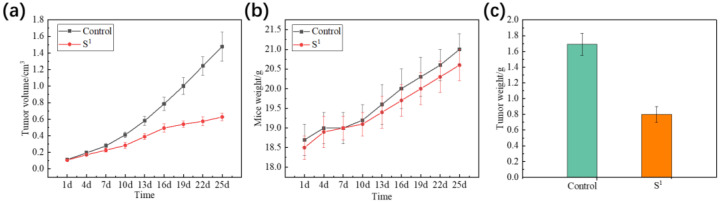
(**a**) Change in the tumor volume of mice injected with S^1^ compared to PBS control; (**b**) change in body weight of mice injected with S^1^ compared to PBS control; (**c**) the tumor weight of mice injected with S^1^ compared to PBS control after 25 days. The dosage of S^1^ was 15 mg/kg.

**Figure 6:**
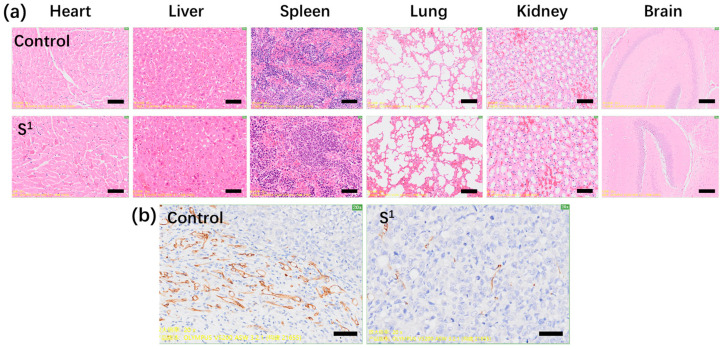
(**a**) Hematoxylin and eosin staining of the brain, heart, liver, spleen, lung, and kidney tissues collected from mice on the 25th day after intravenous injection of S^1^ compared to mice after intravenous injection of PBS on the 25th day as a control; (**b**) CD31 immunohistochemical staining of tumor mice on the 25th day after intravenous injection of S^1^ and PBS control. Scale bar = 20 μm. Error bars are based on standard errors of the mean (*n* = 3). The dosage of S^1^ was 15 mg/kg.

According to Figure 5a–c, the weight and volume of the tumors in the mice reduced after being injected with S^1^ (15 mg/kg) compared with the PBS control. For S^1^, the average volume of the HepG2 tumor was 0.627 cm^3^ and the PBS control was 1.478 cm^3^. Tumor weight was 0.80 g and the weight of the PBS control was 1.69 g. The relative tumor proliferation rate (T/C) was 44.67% and the tumor inhibition rate was 52.76%. Figure 6a shows no obvious changes in the heart, liver, spleen, lung, kidney, or brain tissue morphology of the mice injected with S^1^, suggesting that S^1^ had no significant toxicity. As shown in Figure 6b, tumor mice were immunohistochemically stained with CD31 on the 25th day. The tumor angiogenesis rate decreased in contrast to the PBS control, suggesting that S^1^ could inhibit tumor growth. These data demonstrate that S^1^ had high anti-HepG2 activity both in vitro and in vivo, proving that S^1^ has nontoxic side effects as a potential antiproliferative agent and may be a good choice for future clinical treatment.

#### 3.4.6. DNA Binding Modes

Intercalations affect DNA properties strongly and are reported as preliminary step mutations. Many drugs, such as DOX, can induce apoptosis by embedding or binding to DNA [33]. We investigated whether S^1^ has DNA-binding activity similar to that of DOX. We assessed the binding modes of salmon sperm DNA (fDNA) binding modes using ethidium bromide (EB) fluorescence displacement assays. EB did not emit significant luminescence in the buffer. However, after adding DNA, the fluorescence intensity significantly increased due to the strong insertion of DNA base pairs. The compound’s intercalation into the DNA base pair was confirmed when fDNA-EB emission was reduced or quenched after adding the compound [34,35,36].

The emission intensity was significantly reduced by adding S^1^ to fDNA-EB, as shown in Figure S2, suggesting that S^1^ binds to DNA at sites occupied by EB and interacts with DNA via intercalation. The above tests demonstrated that S^1^ interacted with DNA in an intercalation mode, which changed or destroyed DNA to induce apoptosis and inhibit cell proliferation. Therefore, S^1^ has high antiproliferative activity.

## 4. Discussion

The high nutritional value of blackberries, which includes soluble solids, organic acids, the solidity–acid ratio, and total anthocyanin content, as well as their unique flavor, make them a popular food choice among consumers. Previous studies have demonstrated significant differences in the accumulation of bioactive substances in fruit from different blackberry cultivars [37]. Shuofeng blackberry is a new blackberry variety selected from the F_1_ hybrids “Kiowa” (♀) and “Hull” (♂) that has complementary advantages inherited from its parents. Shuofeng exhibits excellent growth characteristics, including thornlessness, good erectness, and strong growth potential, in addition to its obvious advantages, which include a higher yield, large fruit size, and a longer maturation period. It is about 20–30% taller than the Hull variety and can obtain 10% higher yield. The fruits are black, shiny, productive, and large, with an average single fruit weight of 7.0–8.0 g, representing the largest variety of thornless blackberries. In China, Shuofeng is promoted as an advantageous thornless blackberry variety.

In this work, both Shuofeng anthocyanin and C3G demonstrated cytotoxic activity against Hela, HepG2, MCF-7, and A549 cells. Comparatively, S^1^ had higher cytotoxic activity than C3G because S^1^ is a combination of cyanidin and pelargonidin, with each anthocyanin type having different levels of cytotoxic activity and the combination overall enhancing the antiproliferative activity, which is higher than that of the individual anthocyanins. For example, Li et al. determined that the proliferation of Hela cells treated with 5 µg/mL DDP, 400 µg/mL C3G, or a combination of both 5 µg/mL DDP and 400 µg/mL C3G was inhibited by 17.43, 34.98, and 63.38%, respectively. The IC_50_ value for DDP treatments in Hela cells was 18.53 µg/mL, similar to our result of 18.50 µg/mL [24]. Importantly, C3G enhanced the sensitivity of HeLa cells to cisplatin. Alsharairi extracted and combined five anthocyanins (delphinidin, peonidin, petunidin, cyanidin, and malvidin) from bilberry and blueberry. Their results demonstrated that they inhibited growth and metastasis, induced apoptosis, and arrested the cell cycle of non-small cell lung cancer cells, such as A549 cells, in vitro [25]. He et al. reviewed 30 plant-derived products with antitumor effects on cervical cancer that showed possible benefits in treating patients through various mechanisms, such as apoptosis induction and inhibiting cell proliferation [22]. Li et al. found a combination of C3G and DDP significantly inhibited the activities of SOD, catalase, and glutathione peroxidase by modulating the nuclear factor E2-related factor 2 (Nrf2) signaling pathway to induce oxidative stress and apoptosis in HeLa cells [38]. Apoptosis is a biochemical process in which a group of specific proteins interacts with one another to transmit programed death-inducing signals and decompress cells and involves activating, expressing, and regulating a series of genes. It plays a central role in cancer, as its induction into cancer cells is the key to successful treatment [39]. S^1^ could inhibit cell proliferation by inducing apoptosis, induce ROS production in HepG2 cells, and lead to cell death. ROS can represent a new standard for determining cytotoxicity because increasing intracellular ROS interferes with the oxidative environment of cells and induces cell death [32]. In vivo experiments have indicated that anthocyanins can prevent and treat cancer, with the relative tumor proliferation rate (T/C) being 44.67% and the tumor inhibition rate being 52.76%, similar to our previous paper covering the anticancer activity of natural products and suggesting that it has excellent antiproliferative effects [29,35]. The further mechanism might be that S^1^ can induce apoptosis by interacting with DNA in an intercalation mode, changing or destroying DNA. Lin et al. reviewed the effects of anthocyanins on the prevention and treatment of cancer, showing that they can inhibit proliferation by modulating signal transduction pathways, inducing cell cycle arrest, and stimulating apoptosis or autophagy in cancer cells, as well as reverse drug resistance in cancer cells and increasing their sensitivity to chemotherapy treatments [26].

There are a few limitations in this study. Firstly, we found that the systemic bioavailability of anthocyanins was only 0.26–1.8% in animal studies when compared with intravenous administration [40]. Anthocyanins are rapidly metabolized into several active metabolites by the host and/or gut microbiota and evaluating their bioavailability is complex. This finding implies that a high dose was administered intravenously. The low bioavailability of anthocyanins causes the antiproliferative effect of anthocyanin extract to decrease in vivo. Secondly, the metabolism of anthocyanins remains unclear. Therefore, clinical trials are needed to validate the potential anticancer activities of anthocyanins before clinical use.

## 5. Conclusions

Our results suggest that *Rubus* Shuofeng has a high anthocyanin content and that the main anthocyanin components are cyanidin and pelargonidin: glycosylated or acylated forms of glucose, galactose, and arabinose. Its anthocyanin extract has high antiproliferative effects both in vitro and in vivo, with these effects being especially prominent on HepG2 cells. Shuofeng anthocyanin may be a potential antiproliferative food or drug agent. The mechanism may be that the anthocyanin content in the Shuofeng variety interacts with DNA or alters or destroys DNA in an intercalated manner, causing apoptosis and inhibiting cell proliferation. Research on other possible mechanisms is needed. This work provides a theoretical basis for developing blackberry’s additional value. In the future, we hope to develop target drugs from natural foods to benefit human health.

## Figures and Tables

**Table 1:** Main nutrients in different kinds (lines) of fresh blackberry fruit.

Name	Soluble Solid (%)	Total Acid (Citrate, %)	Solidity–Acid Ratio	Total Anthocyanins (mg/g)
Hull	11.8 ± 0.6	1.0 ± 0.1	12.3 ± 0.9	1.08 ± 0.01
Chester	9.7 ± 0.2	1.24 ± 0.03	7.87 ± 0.09	2.01 ± 0.02
Choctaw	9.5 ± 0.1	0.75 ± 0.03	12.7 ± 0.5	2.24 ± 0.02
Brazos	7.9 ± 0.4	1.0 ± 0.1	7.9 ± 0.5	1.89 ± 0.01
Commanche	10.4 ± 0.4	0.72 ± 0.08	15 ± 1	2.10 ± 0.04
Arapaho	11.7 ± 0.2	0.91 ± 0.03	12.8 ± 0.3	2.08 ± 0.01
Kiowa	11.8 ± 0.2	0.84 ± 0.09	14 ± 1	2.18 ± 0.01
Shawnee	10.1 ± 0.4	0.91 ± 0.09	11 ± 1	2.25 ± 0.03
Natchez	10.8 ± 0.1	1.01 ± 0.06	10.7 ± 0.5	2.05 ± 0.04
Ningzhi‘2’	12.3 ± 0.6	0.81 ± 0.06	15.3 ± 0.5	1.82 ± 0.02
Ningzhi‘3’	10.73 ± 0.06	0.88 ± 0.04	12.2 ± 0.5	1.94 ± 0.03
Shuofeng	10.7 ± 0.2	0.99 ± 0.07	10.9 ± 0.6	2.54 ± 0.01
Zaohei	10.3 ± 0.9	1.2 ± 0.1	8.4 ± 0.4	2.04 ± 0.02
Shuofeng‘2’	7.9 ± 0.4	0.89 ± 0.04	8.88 ± 0.05	1.97 ± 0.03
10-5n-2	10.8 ± 0.4	1.05 ± 0.09	10.3 ± 0.7	2.06 ± 0.03
15-1-1	13.2 ± 0.7	0.83 ± 0.05	15.8 ± 0.1	2.29 ± 0.03
15-1-2	12.6 ± 0.6	1.15 ± 0.08	11.0 ± 0.3	2.29 ± 0.02
15-1-3	11.1 ± 0.4	0.98 ± 0.07	11.4 ± 0.4	2.11 ± 0.04
15-1-6	10.8 ± 0.3	0.77 ± 0.02	14.0 ± 0.1	2.06 ± 0.02
15-1-9	12.0 ± 0.3	1.01 ± 0.02	11.8 ± 0.2	2.01 ± 0.02
15-1-10	12.5 ± 0.5	0.95 ± 0.03	13.2 ± 0.1	1.95 ± 0.03
15-1-11	14.4 ± 0.4	1.1 ± 0.1	13 ± 1	2.18 ± 0.01
15-1-17	11.6 ± 0.3	0.72 ± 0.04	16.2 ± 0.6	1.63 ± 0.02
16n-12	12.6 ± 0.1	0.75 ± 0.02	16.9 ± 0.4	2.35 ± 0.04
C28-4	12.5 ± 0.5	0.9 ± 0.1	14 ± 1	1.93 ± 0.01
Average	11.13	0.94	12.28	2.04

**Table 2:** UPLC-QTOF-MS identification of anthocyanin components in blackberries.

No.	RT/min	MS/[M]^+^	MS^2^	Content/%	Tentative Identification
1	19.27	449.1	287.1	26.67	Cya-glc
2	21.50	419.1	287.1	0.74	Cya-ara/xyl
3	27.53	419.1	287.1	5.19	Cya-ara/xyl
4	29.58	535.1	287.1	8.88	Cya-malonyl-glc
5	30.60	593.1	287.1	7.41	Cya-dioxalyl-glc
6	32.47	519.2	271.1	5.93	Pel-malonyl-glc
7	32.83	519.2	271.1	23.70	Pel-malonyl-glc
8	33.94	563.1	287.1	18.52	Cya-dioxalyl-ara/xyl
9	34.19	621.2	287.1	2.96	Cya-unknown

Cya: cyanidin; Pel: pelargonidin; glc: glucoside; gal: galactoside; ara: arabinoside; xyl: xyloside.

**Table 3:** Antiproliferative activities of S^1^, DDP, DOX, S^1^-DDP, S^1^-DOX, and C3G (IC_50_ ± SE *^a^*/μgmL^−1^).

Compd.	Hela	HepG2	MCF-7	A549	HUVEC
S^1^	68.62 ± 3.78	55.85 ± 3.56	181.21 ± 4.28	82.01 ± 3.93	115.43 ± 1.68
S^1^-DDP	32.70 ± 1.3 **^b^	31.78 ± 1.56 *^c^	118.13 ± 4.43 ***^a^	68.47 ± 2.40 **^b^	38.74 ± 1.25 *^c^
S^1^-DOX	2.88 ± 0.39 ***^a^	0.08 ± 0.003 ***^a^	0.80 ± 0.03 ^ns*d*^	0.44 ± 0.03 *^c^	8.47 ± 0.27 ^ns*d*^
DDP	18.50 ± 1.23 *^c^	18.90 ± 0.72 **^b^	39.88 ± 1.50 ***^a^	51.92 ± 4.48 ***^a^	26.46 ± 1.10 **^b^
DOX	0.96 ± 0.13 ***^a^	0.32 ± 0.13 ***^a^	0.65 ± 0.10 *^c^	0.10 ± 0.01 ^ns*d*^	0.58 ± 0.07 ^ns*d*^
C3G	126.31 ± 5.79 **^b^	147.71 ± 9.16 ^ns*d*^	239.05 ± 3.99 **^b^	182.24 ± 4.10 **^b^	205.14 ± 4.05 *^c^

*^a^* Average IC_50_ values from at least three independent experiments. ***^a^ p < 0.001; **^b^ p < 0.01; *^c^ p < 0.05; ^ns*d*^ = not significant, compounds compared with S^1^, respectively.

## Data Availability

Data are available upon request.

## Supplementary Materials

The following supporting information can be downloaded at: https://www.mdpi.com/article/10.3390/foods12061216/s1, Figure S1: Liquid chromatograph of extract from “Shuofeng” at 520nm; Figure S2: Emission spectra of fDNA-EB in the absence and presence of S1 at room temperature, respectively ([EB] = 2 × 10^−5^ M, [fDNA] = 1 × 10^−4^ M, and [S^1^] = 1.5 × 10^−5^ M).

## Abbreviations

Hela, cervical cancer cells; HepG2, hepatoma cells; MCF-7, breast cancer cells; A549, non-small cell lung cancer cells; HUVEC, normal human umbilical vein endothelial cells; MTT, 3-(4,5-dimethylthiazol-2-yl)-2,5-diphenyltetrazolium bromide; DMEM, Dulbecco’s Modified Eagle’s Medium; FBS, fetal bovine serum; DDP, cis-platinum; DOX, doxorubicin; C3G, cyanidin 3-O-glucoside; TIMP-1, tissue inhibitor of metalloproteinase-1; PDGF, platelet-derived growth factor; AP-1, activator protein 1; VEGF, vascular endothelial growth factor; JNK, c-JUN NH2-terminal kinase; p53 MAPK, mitogen-activated protein kinase; S^1^, “Shuofeng” anthocyanin extract; DCFH-DA, dihydrodichlorofluorescein diacetate; NAC, N-acetylcysteine; ROS, reactive oxygen species.
