# Supplementary material for: Research on Anthocyanins from Rubus “Shuofeng” as Potential Antiproliferative and Apoptosis-Inducing Agents
Source: Foods. 2023 Mar 13;12(6):1216. doi: 10.3390/foods12061216 (PMC10048323; doi:10.3390/foods12061216)
Supplement: Supplementary file 1 [file foods-12-01216-s001.zip › foods-2252997-supplementary.pdf]

Supplementary material

Research on Anthocyanins from *Rubus* "Shuofeng" as Potential Antiproliferative and Apoptosis-Inducing Agents

Fengyi Zhao [1], Huifang Zhao [1], Wenlong Wu [1,*], Weifan Wang [2,*] and Weilin Li [3]

1 Fruit Research Center, Institute of Botany, Jiangsu Province and Chinese Academy of Sciences, Nanjing 210014, China
2 College of Chemical Engineering, Nanjing Forestry University, Nanjing 210037, China
3 Co-Innovation Center for the Sustainable Forestry in Southern China, College of Forestry, Nanjing Forestry University, Nanjing 210037, China; wlli@njfu.edu.cn

* Correspondence: 1964wwl@163.com (W.W.); weifan@njfu.edu.cn (W.W.); Tel.: +86-2584347046 (Weifan Wang)

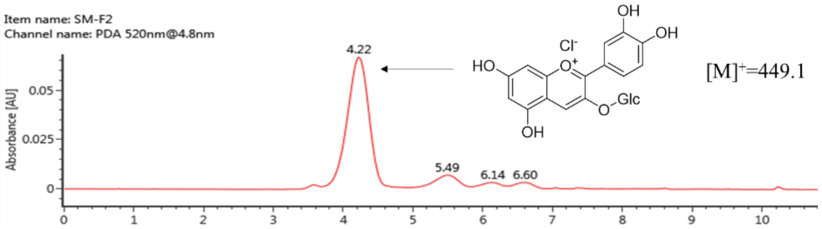

Figure S1. Liquid chromatograph of extract from 'Shuofeng' at 520nm.

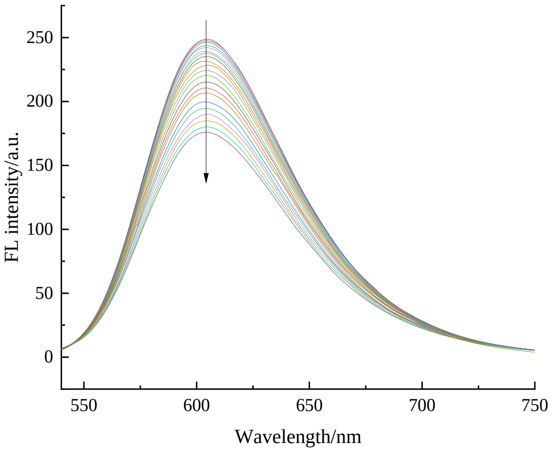

Figure S2. Emission spectra of fDNA-EB in the absence and presence of increasing amounts of S^1 at room temperature, respectively ([EB] = 2×10^{-5} M, [fDNA] = 1×10^{-4} M, and [S^1] = 1.5×10^{-5} M).
